# Land Use Change and Climate Variation in the Three Gorges Reservoir Catchment from 2000 to 2015 Based on the Google Earth Engine

**DOI:** 10.3390/s19092118

**Published:** 2019-05-07

**Authors:** Binfei Hao, Mingguo Ma, Shiwei Li, Qiuping Li, Dalei Hao, Jing Huang, Zhongxi Ge, Hong Yang, Xujun Han

**Affiliations:** 1 Research Base of Karst Eco-Environments at Nanchuan in Chongqing, Ministry of Nature Resources, Chongqing Key Laboratory of Karst Environment, School of Geographical Sciences, Southwest University, Chongqing 400715, China; hbfnihao@email.swu.edu.cn (B.H.); mmg@swu.edu.cn (M.M.); li41680@outlook.com (Q.L.); jingjing520@email.swu.edu.cn (J.H.); zhongxige@outllook.com (Z.G.); 2 Chongqing Engineering Research Center for Remote Sensing Big Data Application, School of Geographical Sciences, Southwest University, Chongqing 400715, China; 3 Chongqing Jinfo Mountain Field Scientific Observation and Research Station for Karst Ecosystem, School of Geographical Sciences, Southwest University, Chongqing 400715, China; 4 Shang Zheng (Beijing) Information Technology Co., Ltd., Beijing 100086, China; lishiwei@agrisz.com; 5 State Key Laboratory of Remote Sensing Science, Institute of Remote Sensing and Digital Earth, Chinese Academy of Sciences, Beijing 100101, China; haodl@radi.ac.cn; 6 Department of Geography and Environment Science, University of Reading, Reading RG6 6AB, UK

**Keywords:** Land use and land cover change (LULCC), land surface temperature (LST), seasonally integrated normalized difference vegetation index (SINDVI), Climate change, Three Gorges Reservoir Catchment (TGRC), Google Earth Engine

## Abstract

Possible environmental change and ecosystem degradation have received increasing attention since the construction of Three Gorges Reservoir Catchment (TGRC) in China. The advanced Google Earth Engine (GEE) cloud-based platform and the large number of Geosciences and Remote Sensing datasets archived in GEE were used to analyze the land use and land cover change (LULCC) and climate variation in TGRC. GlobeLand30 data were used to evaluate the spatial land dynamics from 2000 to 2010 and Landsat 8 Operational Land Imager (OLI) images were applied for land use in 2015. The interannual variations in the Land Surface Temperature (LST) and seasonally integrated normalized difference vegetation index (SINDVI) were estimated using Moderate Resolution Imaging Spectroradiometer (MODIS) products. The climate factors including air temperature, precipitation and evapotranspiration were investigated based on the data from the Global Land Data Assimilation System (GLDAS). The results indicated that from 2000 to 2015, the cultivated land and grassland decreased by 2.05% and 6.02%, while the forest, wetland, artificial surface, shrub land and waterbody increased by 3.64%, 0.94%, 0.87%, 1.17% and 1.45%, respectively. The SINDVI increased by 3.209 in the period of 2000-2015, while the LST decreased by 0.253 °C from 2001 to 2015. The LST showed an increasing trend primarily in urbanized area, with a decreasing trend mainly in forest area. In particular, Chongqing City had the highest LST during the research period. A marked decrease in SINDVI occurred primarily in urbanized areas. Good vegetation areas were primarily located in the eastern part of the TGRC, such as Wuxi County, Wushan County, and Xingshan County. During the 2000–2015 period, the air temperature, precipitation and evapotranspiration rose by 0.0678 °C/a, 1.0844 mm/a, and 0.4105 mm/a, respectively. The climate change in the TGRC was influenced by LULCC, but the effect was limited. What is more, the climate change was affected by regional climate change in Southwest China. Marked changes in land use have occurred in the TGRC, and they have resulted in changes in the LST and SINDVI. There was a significantly negative relationship between LST and SINDVI in most parts of the TGRC, especially in expanding urban areas and growing forest areas. Our study highlighted the importance of environmental protection, particularly proper management of land use, for sustainable development in the catchment.

## 1. Introduction

Global climate change and human activities have largely affected the earth’s surface and ecosystem on different scales. In particular, land use/land cover change (LULCC) directly reflects the impact of land surface process [1,2], and it also affects the biogeochemical process and ecosystem function, such as soil erosion, water cycling, carbon cycling and biodiversity [3,4]. Therefore, knowledge of LULCC is crucial for understanding the relationship between human social and economic activities and natural ecological processes [5]. 

There are different approaches to research LULCC. For example, historical documents have been used as the main data sources for estimating the global LULCC over the past 300 years [6]. Ancient atlases were compiled to study the LULCC in Southern Germany during the 17th~19th centuries [7]. Historical field survey data were also applied to research the LULCC in Wisconsin, the USA from 1850 to 2000 [8]. More importantly, multisource information, such as in situ survey and Remote Sensing (RS) were synthesized to study the LULCC at global and regional scales [9]. It is worth noting that RS has accelerated the analyses of the landscape dynamics [10]. Many studies have been devoted to land use mapping, change detection and vegetation dynamics monitoring using multi-temporal satellite RS data for multi-scale ecological and environmental researches [11,12,13,14]. A series of approaches have been developed for land use classification and change detection, for example, pixel-based classification [15], object-oriented classification [16], artificial neural network classification [17], visual interpretation [18] and classification and regression trees [19]. Moreover, a quite important remote sensing concept known as the normalized difference vegetation index (NDVI) has been developed and applied in many studies on LULCC, including vegetation phenology and dynamics at annual and inter-annual levels [20,21,22,23,24]. For example, the afforestation supported by the Chinese government resulted in an increase in NDVI [25], while rapid urban expansion has an opposite impact. LULCC also plays an important role of influencing land surface temperature (LST) [26]. Urban areas covered by buildings, roads and other impervious surfaces generally have higher solar radiation absorption and a greater thermal capacity and conductivity. Therefore, urban areas tend to experience a relatively higher temperature compared with the surrounding rural areas [27]. In comparison, the farmland, forest, grass and waterbody have lower LST [28]. In addition, LST is influenced by meteorological and hydrological conditions [27,29,30]. 

One of the land use types receiving increasing attention is artificial waterbodies, for example hydropower reservoirs. As the largest hydroelectric project in the world, Three Gorges Project (TGP) has provided major social and economic benefits, for example flood control, electricity generation and navigation. However, the potential impacts on the natural environment cannot be ignored [31,32]. The Three Gorges Reservoir Catchment (TGRC) has a large influence on the environment, ecosystem and people’s lives in the area and outside of it. Soil erosion, water pollution, loss of biological diversity (e.g., *Adiantum reniforme Linnaeus* var. *sinense*, *Myricaria laxiflora* and *Acipenser sinensis*), and resettlement of people in certain parts of the TGRC have been alarming problems [33,34]. An additional impact of TGP is the large LULCC due to rising water level, population migrations, and urban expansion after the construction of TGP [35,36,37]. Along with impacts on vegetation coverage [38], LULCC affects LST at a large extent [39]. Meanwhile, it also influences climate change locally or at a large scale [40]. Therefore, determining the LULCC, vegetation coverage, LST and climate variation in the TGRC is important for a better understanding of how and to what extent does LULCC affect these variables. 

To analyze the spatiotemporal dynamics of the LULCC, LST, NDVI and climate change, satellite RS has been proven to be an important data source. There is a new tech-tool, Google Earth Engine. As a cloud-based platform for planetary-scale geospatial analysis, it brings Google’s massive computational capabilities to bear on a variety of high-impact societal issues including vegetation degradation, drought, water resource management, climate monitoring and environmental protection [41]. Powered by Google’s cloud infrastructure, it combines a large number of geospatial datasets and satellite imagery in which the datasets can be processed, analyzed and visualized at local, regional and global scales [42]. 

Several studies have researched the LULCC in the TGRC. However, most studies have only covered periods before 2010 [43,44], and the recently detailed land use structure in the TGRC is largely lacking. Moreover, most studies on NDVI changes in the TGRC were before 2011 [45,46] and the recent vegetation change is largely unknown. There are some studies on LST in the TGRC. However, they focused on retrieval algorithm and urban heat island (UHI) in certain parts of the TGRC, such as the Chongqing section, the middle reaches of the TGRC, and for a short research period [47,48]. Moreover, the relationship of climate change between the TGRC and Southwest China was investigated in order to explore the causes and extent of climate change in the TGRC. Most studies only carry out unilateral research analysis without a comprehensive understanding of LULCC, LST, vegetation cover and climate variation. Therefore, it is still necessary to explore the influence of LULCC on these factors. What is more, there is no study that uses the state-of-the-art GEE platform to track and monitor the LULCC and its impacts on LST, vegetation cover and climate variation in the TGRC. 

The GEE provides a consolidated environment including a large amount of data catalogue co-located with thousands of computers for analysis [49,50]. First, we collected the samples and developed the classification algorithm and then imported it into GEE to test and validate the classification results (in 2015) with previous studies. Second, we developed the processing algorithms of Moderate Resolution Imaging Spectroradiometer (MODIS) LST and NDVI and then deployed them in GEE to obtain the slope, range and correlation analysis results of them. Third, we conducted an algorithm for Global Land Data Assimilation System (GLDAS-2) in GEE and then obtained the annual results. Finally, we obtained all the study results in GEE platform instead of local processing. To the best of our knowledge, the current study made the first attempt to use the new method/platform, GEE, to research the environmental change in the TGRC. 

Environmental changes in the TGRC have important impacts on human society and the ecosystem. Despite of some studies on environmental change in the TGRC, recent environmental changes in the region are is still largely unknown, particularly during the last five years. Our study about vegetation cover, LST and climate change affected by LULCC can provide timely suggestions for environmental protection and sustainable development of the TGRC. In this study, 30 m global land cover datasets (GlobeLand30) were analyzed. In addition, Landsat Operational Land Imager (OLI) data, the MODIS datasets including the LST product (MOD11A2) and vegetation indices product (MOD13A2), as well as the GLDAS-2 data (precipitation, air temperature and evapotranspiration), which were archived in the GEE, were investigated. The objectives of this study are: (1) to analyze the LULCC in the TGRC from 2000 to 2015; (2) to estimate the dynamics of LST, SINDVI, and climate; and (3) to analyze the impacts of LULCC on LST, SINDVI and climate change. 

## 2. Material and Methods

### 2.1. Study Area

**Figure 1:**
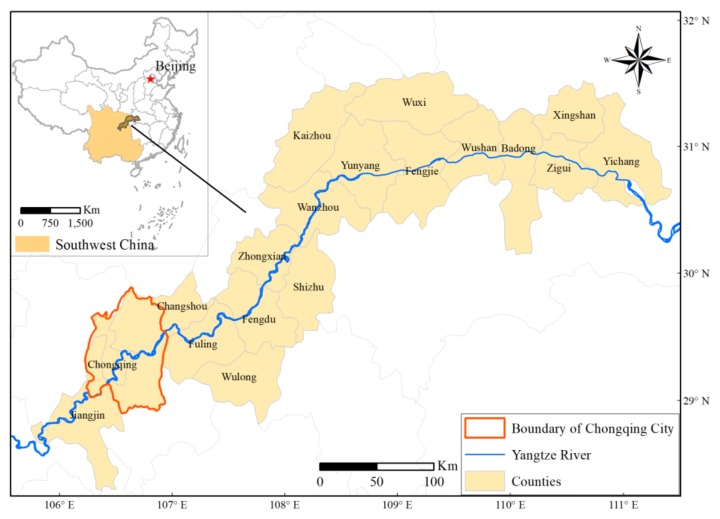
The study area and its location in Southwest China.

The Three Gorges Reservoir Catchment (TGRC) (28°56′N-31°44′N, 106°16′E-111°28′E) is located in the lower section of the upper reaches of the Yangtze River, Southwest China. The total area is approximately 58,000 km^2^ and the local population is nearly 30 million. It consists of 21 counties, districts or cities associated with the Chongqing municipality and the Hubei province (Figure 1). The elevation ranges from 78 to 3061 m. Mountains (altitude ≥ 500 m, mostly in the northeast and south bank of Yangtze River), hills (200 m ≤ altitude < 500 m, mostly in the mid-west) and plains (altitude < 200 m) account for approximately 74.0%, 21.7% and 4.3% of total area [46,51]. It has a subtropical monsoon climate with the average annual precipitation of 1000~1800 mm and average annual air temperature of 14.9~18.5 ℃ [52]. Approximately 6388 higher plants, 523 terrestrial vertebrates, 3481 insects, and 350 fish have been found in the area [2]. The water level of the TGRC started to reach 135.0 m in 2003, 156.0 m in 2006, and 175.0 m in 2010. 

### 2.2. Data Collection and Processing

The world’s first 30 m global land cover datasets from GlobeLand30 with an overall accuracy of 83.5% [53] meet the requirement of the LULCC research in the TGRC and were used in this study. However, the GlobeLand30 only has two base year products of 2000 and 2010. In order to extend the time-series research of the LULCC, the Landsat 8 OLI images from 2015 and 30-m digital elevation data from the Shuttle Radar Topography Mission (SRTM) were analyzed. The MODIS data including the LST product and vegetation index product were applied. The GLDAS-2 has two datasets including GLDAS-2.0 and GLDAS-2.1, and the GLDAS-2.1 simulation started on 1 January, 2000 using the conditions from the GLDAS-2.0 simulation [54]. The GLDAS-2 data including the precipitation, temperature and evapotranspiration were used, and the data quality of GLDAS-2 is better than that of GLDAS-1 in China [55,56,57]. Except for the GlobeLand30 data, the rest of the data employed in this study were obtained from the GEE, and they were scientifically organized and managed to facilitate fast and efficient access. The data archived in the GEE were originally provided by the United States Geological Survey (USGS), the NASA Jet Propulsion Laboratory (JPL-Caltech), the Land Processed Distributed Active Archive Center (LP DAAC) managed by the NASA. 

**Figure 2:**
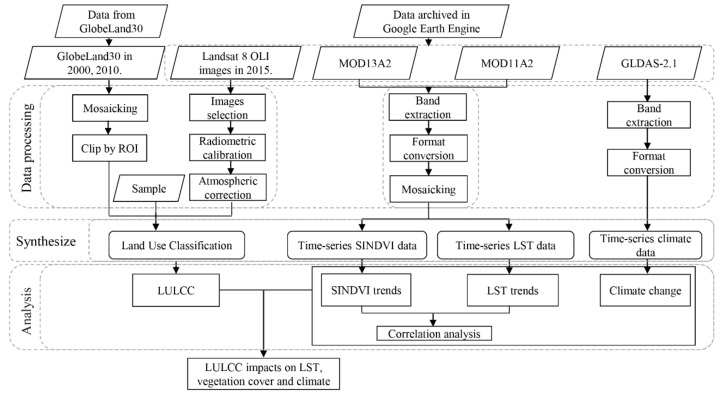
Workflow of the study. GLDAS: Global Land Data Assimilation System; ROI: region of interest; LULCC: land use and land cover change; SINDVI: seasonally integrated normalized difference vegetation index; LST: land surface temperature.

The Google Earth Engine provides online access to its archived datasets. In this study, the JavaScript application programming interface (API) was used for calling, preprocessing, mosaicking, and processing the Landsat 8 OLI, MODIS LST and NDVI data. Subsequently, the objective data were generated and then exported using the coordinate reference system from World Geodetic System 1984 (WGS84). GLDAS-2.1 data were processed using the JavaScript API (Figure 2). Consequently, the land use types in 2000, 2010 and 2015 were analyzed using ArcGIS 10.2 (ESRI, Redland, CA, USA) and ENVI software (Exelis Visual Information Solutions, Boulder, CO, USA). The NDVI, LST and GLDAS-2.1 data were analyzed using GEE platform, Origin2017 (OriginLab, Northampton, Massachusetts, USA) and ArcGIS software. 

#### 2.2.1. GlobeLand30 Land Use Data

The GlobeLand30 product was developed by China under the support of 863 key projects and administered by the National Geomatics Center of China (NGCC) [53,58]. It contains ten major land classes: cultivated land, forest, grassland, shrub land, wetland, waterbody, tundra, artificial surface, bare land and permanent snow and ice, from the base years 2000 and 2010, with the spatial resolution of 30 m [58]. In the TGRC, the land use types consist of seven classes, namely, cultivated land, forest, grassland, shrub land, wetland, waterbody and artificial surface.

#### 2.2.2. Land Use Data Derived from Landsat 8 OLI Data

**Figure 3:**
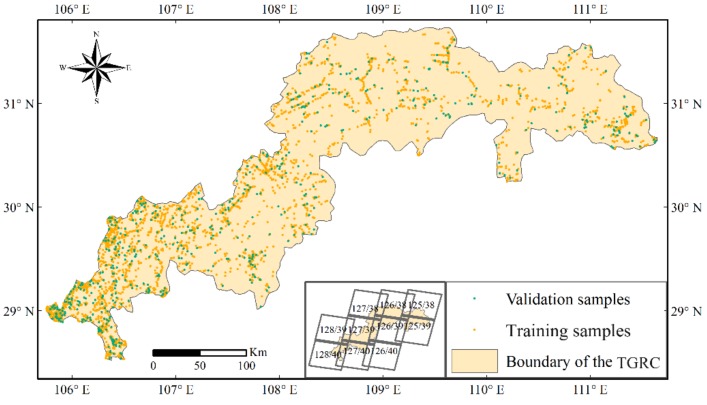
Training and validation sample sites in the TGRC.

The TGRC is located within ten scenes (path/row: 125/38, 125/39, 126/38, 126/39, 126/40, 127/38, 127/39, 127/40, 128/39, and 128/40) in World Reference System-2 (WRS-2) (Figure 3). To extend the time series analysis of the LULCC, this study used Landsat 8 OLI-calibrated top-of-atmosphere (TOA) reflectance images under cloudy coverage lower than 10% from 2015 to interpret the land use result through visual interpretation. The training and validation samples were manually interpreted by referring to the high-resolution time series images in Google Earth. Seven land use types were identified according to the Globeland30 land use types in the TGRC. 

The Classification and Regression Tree (CART) was chosen as a classifier. First, samples (Figure 2) consisting of 2484 training samples and 1064 validation samples (Table 1) were uploaded to the GEE via Google Fusion Table. Second, Landsat 8 OLI image-related bands [59] (Table 2) were used to compute the NDVI, the Normalized Difference Water Index (NDWI) and the Normalized Difference Built-up Index (NDBI) due to their important discriminating values when classifying the type of forest, grassland, building and waterbody [60,61]. Third, digital elevation data were used to assist with the classification. Finally, the post-classification, such as a majority analysis, was used to eliminate noise from the classification result and improve the quality of the classification map. The NDVI, NDWI and NDBI were calculated as follows:

(1) $$\begin{array}{c} NDVI=\;\frac{NIR-Red}{NIR+Red}\;\;\;, \end{array}$$

(2) $$\begin{array}{c} NDWI=\;\frac{Green-NIR}{Green+NIR}\;\;, \end{array}$$

(3) $$\begin{array}{c} NDBI=\;\frac{SWIR1-NIR}{SWIR1+NIR}\;\;\;, \end{array}$$

where *NIR* is the reflectance of Band 5, *Red* is the reflectance of Band 4, *Green* is the reflectance of Band 3, and *SWIR1* is the reflectance of Band 6 on the Landsat 8 OLI images. 

#### 2.2.3. Normalized Difference Vegetation Index

This study used the 16-day composite vegetation index product (MOD13A2, Collection 6) with resolution of 1000 m, which was synthesized by selecting the best available pixel value from all the acquisitions taken over a 16-day period. Data between February 2000 and December 2015 were used. 

In addition, the MODIS NDVI time series data were still influenced by the clouds and atmospheric water vapor [22,23], a simple but efficient method based on a mean-value iteration filter was used to reduce the noise and improve the data quality by using the following equation [22]:

(4) $$\begin{array}{c} \Delta_{i}=\;\left|NDVI_{i}\right.-\left.\left(NDVI_{i-1}+NDVI_{i+1}\right)/2\right|\;, \end{array}$$

where *i* is the *i*th monthly NDVI (*i* varies from 1 to 191 for the 16 years). The threshold value (Δ) could be set as a small percentage of the multiyear average NDVI for each pixel. When the $\Delta_{i}$ is greater than a threshold value, $NDVI_{i}$ is replaced by$\;\left(NDVI_{i-1}+NDVI_{i+1}\right)/2$. The iteration will end when all the$\;\Delta_{i}$ are less than Δ. 

The seasonally integrated normalized difference vegetation index (SINDVI) was used to characterize the range of vegetation conditions [23]. The SINDVI is described by each pixel’s sum of NDVI values while the NDVI exceeds the threshold value (which is commonly defined as NDVI > 0.1) [62]. The grid cells with the NDVI values greater than 0.1 were used to eliminate the influence of bare and sparsely vegetated regions and to determine the growing season [21].

#### 2.2.4. Land Surface Temperature

The MODIS provides an average 8-day land surface temperature (LST) dataset (MOD11A2, Collection 6) with a resolution of 1000 m, and the LST_Day_1 km band was applied to analyze the variation trend in the LST from February 2000 to December 2015. In addition, Kelvin was converted to Celsius with the following formula:

(5) $$\begin{array}{c} t=a*pixel+b\;, \end{array}$$

where t represents the real LST value (°C), *a* is the scale factor with a value of 0.02, *pixel* is the Digital Number (DN) value, and *b* is the offset with a value of -273.15. 

#### 2.2.5. Meteorological Data

GLDAS is a global land surface simulation system with a spatial resolution of 0.25 degrees. It supplies 28 variables including temperature, precipitation, radiation, soil moisture and evapotranspiration. The temperature, precipitation and evapotranspiration were selected from the GLDAS-2.1 Noah model from 2000 to 2015, with its 0.25-degree spatial resolution and 3 h temporal resolution. The meteorological data were ultimately synthesized into an annual scale. 

### 2.3. Methodology

#### 2.3.1. Time Series Analysis

**Figure 4:**
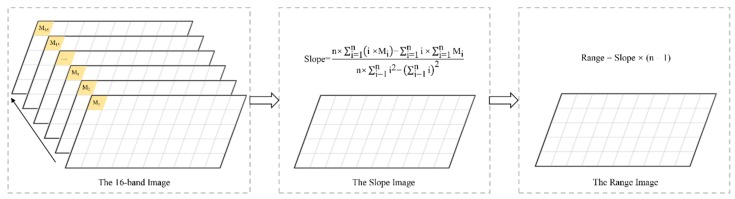
Workflow of the satellite image treatment.

The land use results in 2000, 2010 and 2015 were used to analyze the LULCC. To determine the spatial distribution and temporal variation trends of LST and SINDVI, the monthly time-series LST and NDVI data were processed and synthesized into an annual data, and then the time series in each pixel has a length of 16 (from 2000 to 2015) (Figure 4). In addition, the meteorological data were processed and synthesized into an annual scale to analyze the climate variation from 2000 to 2015 in the TGRC and Southwest China. 

The slope and range of the SINDVI and LST were calculated to estimate their inter-annual variation trends from 2000 to 2015. The slope represents the change rate of each pixel in the period of 2000-2015 calculated by using an ordinary least squares estimation in a linear regression with the follow equation:

(6) $$\begin{array}{c} \mathrm{Slope}=\frac{n\times \sum_{i=1}^{n}\left(i\times M_{i}\right)-\sum_{i=1}^{n}i\times \sum_{i=1}^{n}M_{i}}{n\times \sum_{i=1}^{n}i^{2}-{\left(\sum_{i=1}^{n}i\right)}^{2}}\;,\;\;\; \end{array}$$

where *n* represents the length of the studied years; *i* = 1 is the year 2000, *i* = 2 is the year 2001, and so on; and *M_i_* indicates the SINDVI or LST for *i* year in the region. The “Slope” indicates the trend of the factor in *n* year. The slope of positive value indicates an increasing trend, while the slope with negative value shows a decreasing trend. The greater the slope is, the more obvious the trend becomes. 

In addition, each pixel value indicates the total change of SINDVI and LST during the period of 2000-2015, the range of SINDVI and LST could determine the total spatiotemporal change trend.

(7) $$\begin{array}{c} \mathrm{Range}\;=\;\mathrm{Slope}\;\times \;\left(n\;-\;1\right), \end{array}$$

where *n* represents the number of years.

#### 2.3.2. Correlation Analysis

To determine the relationship between SINDVI and LST, the correlation coefficients between these two variables were calculated for each pixel as follows:

(8) $$\begin{array}{c} r_{XY}=\;\frac{\sum_{i=1}^{n}\left(X_{i}-\bar{X}\right)\left(Y_{i}-\bar{Y}\right)}{\sqrt{\sum_{i=1}^{n}{\left(X_{i}-\bar{X}\right)}^{2}}\sqrt{\sum_{i=1}^{n}{\left(Y_{i}-\bar{Y}\right)}^{2}}}, \end{array}$$

where $r_{XY}$ represents the correlation coefficient between *X* and *Y*, with the range from −1 to 1. *X_i_* and *Y_i_* are the values in *i* year; and $\bar{X}$ and $\bar{Y}$ are the average values of all years. If *r* > 0, it means a positive correlation between *X* and *Y*. If *r* < 0, it means a negative correlation between these two variables. The greater the |*r*| is, the closer of two variables are. Moreover, *P* value we calculated to assess the statistical significance of the correlation analysis. All correlation analyses were conducted using SPSS 19.0 (Statistical Product and Service Solutions, IBM, Armonk, NY, USA). 

## 3. Results

### 3.1. Land Use and Land Cover Changes

**Figure 5:**
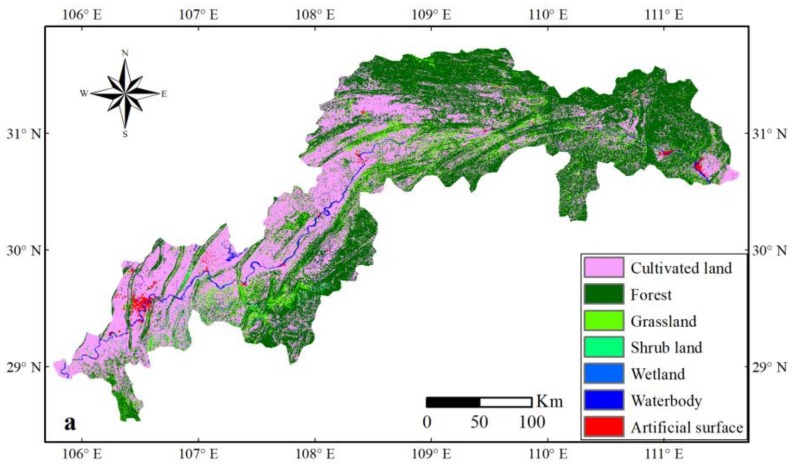

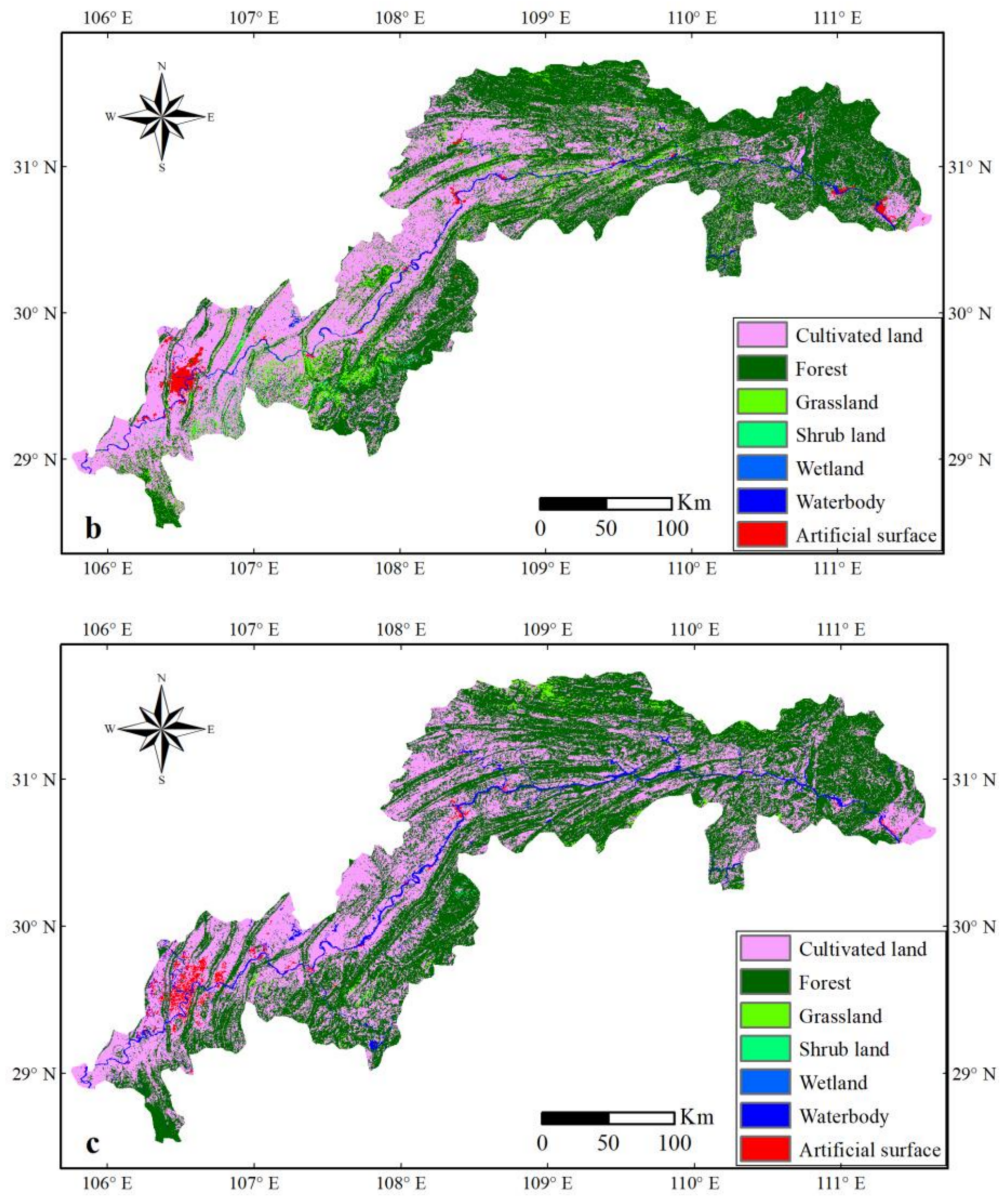
Land use maps in the TGRC in 2000 (**a**), 2010 (**b**) and 2015 (**c**).

The classification and regression tree (CART) was applied to generate the land use map in 2015 with high accuracy, with an overall accuracy of 95.02% and a Kappa index of 0.92. The land use maps in 2000 and 2010 were obtained using the GlobeLand30 from the NGCC. The exact area is listed in Table 3 and Figure 5.

The artificial surfaces including residential, industrial, mining land, and transportation land increased from 509.90 km^2^ to 730.57 km^2^ between 2000 and 2010, at a rate of +4.33%/a, and it increased stably again from 730.57 km^2^ to 1008.96 km^2^ from 2010 to 2015 at a rate of +7.62%/a. The forest increased stably from 26,998.80 km^2^ to 29,102.28 km^2^ between 2000 and 2015, at a rate of +0.52%/a. Waterbodies expanded from 776.10 km^2^ to 1,646.45 km^2^ in the period of 2000-2015, at a rate of +7.22%/a. Wetland decreased from 50.97 km^2^ to 29.33 km^2^ from 2000 to 2010, while it increased rapidly to 591.65 km^2^ from 2010 to 2015 at a rate of +383.44%/a. Cultivated land shrank from 23,727.43 km^2^ to 23,687.01 km^2^ from 2000 to 2010, at a rate of −0.02%/a, and it decreased again to 22,544.68 km^2^ by 2015, at a rate of −0.96%/a. Grassland decreased from 5,293.76 km^2^ to 1820.08 km^2^ in the period of 2000–2015 at a rate of −4.37%/a. The shrub land increased stably from 312.59 km^2^ to 985.45 km^2^ in the period of 2000–2015 with a total increase rate of +14.35%/a. 

There were two main land use types: forest and cultivated land. Forest was primarily distributed in the west side of the head, south of the belly of the TGRC, such as Wulong County, Shizhu County, Wuxi County, Fengjie County, Xingshan County, Zigui County and Yichang City. The cultivated land appeared primarily in the tail, head and the northwest of the Yangtze River, especially the upper reaches in the TGRC, such as Jiangjin County, Chongqing City, Changshou District, Fuling District, Wulong County, Zhong County, Kaizhou District and Yichang City. In addition, the artificial surfaces expanded in the urbanized area, and they were primarily distributed along the head and tail of the TGRC, such as Chongqing City and Yichang City. Moreover, there was a certain amount of artificial surfaces along the Yangtze River of the TGRC, for example, Fuling District and Wanzhou District. 

### 3.2. The Change of Seasonally Integrated Normalized Difference Vegetation Index (SINDVI) 

**Figure 6:**
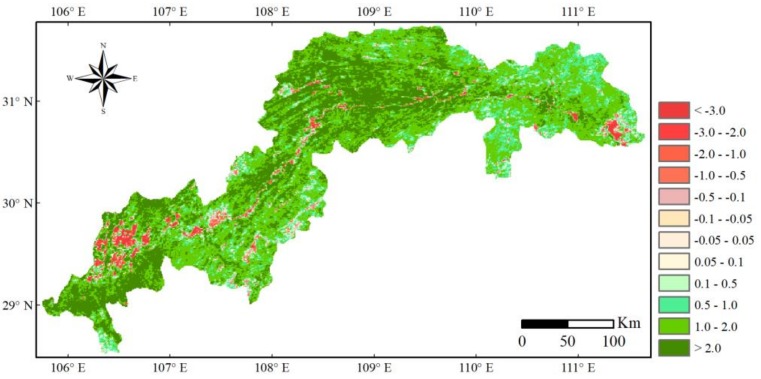
Change of seasonally integrated normalized difference vegetation index (SINDVI) in the TGRC from 2000 to 2015.

The SINDVI showed a greening trend in the TGRC (Figure 6), with a regional average rise of 3.209 from 2000 to 2015, and it was showed a significant increasing trend at 0.1197/a (*p* < 0.01, R^2^ = 0.6176). A statistical table (Table 4) was generated by calculating the area percentage of vegetation change conditions. The results showed that 93.23% of the area experienced an increasing trend (“> 0.1”), 1.24% of the area showed no significant change (“-0.1 – 0.1”) and 4.53% of the area showed a decreasing trend (“< −0.1”). In addition, the vegetation degradation area was primarily distributed along the tail and head of the TGRC, and the rest of the vegetation degradation was primarily located in the center of the TGRC. There was less vegetation in the urbanized areas, such as Chongqing City, Changshou District, Fuling District, Wulong County, Wanzhou District and Yichang City. In addition, there was more vegetation in west of the head and south of the tail of the TGRC, such as Jiangjin County, Yunyang County, Fengjie County, Wuxi County and Wushan County. 

### 3.3. The Change of Land Surface Temperature

**Figure 7:**
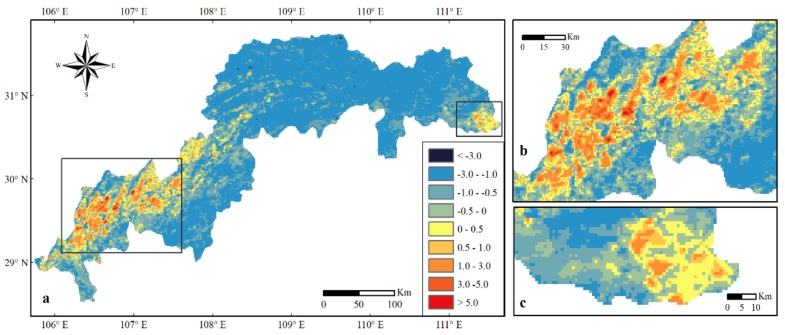
Change of land surface temperature (LST) (°C) in the TGRC in the period of 2000-2015.

There was a decreasing trend of LST at −0.0268 °C/a (*R*^2^ = 0.0849) in the TGRC from 2001 to 2015 (the LST value in 2000 was excluded for its marked increase in the year), with a regional mean decreased of 0.253 °C. A total of 88.76% of the TGRC area showed a decreasing trend of LST (value range < 0), while 11.24% of the TGRC area indicated an increasing pattern (value range > 0). The LST change in the TGRC during the research period is illustrated in Figure 7a. Clearly, a marked increase in the LST appeared in the head (Figure 7b) and tail (Figure 7c) of the TGRC and along the northwestern belly of the TGRC. Moreover, LST in the urban areas, such as Chongqing City, Changshou District, Fuling District (Figure 7b) and Yichang City, which were higher than those in the outskirts of the cities and forests (Figure 7c). The highest temperatures appeared in the Chongqing City region, whereas the lower temperatures were primarily distributed along the eastern part of the TGRC, for example in Kaizhou District, Yunyang County, Wuxi County, Fengjie County, Wushan County, Zigui County, Xingshan County and Badong County. 

### 3.4. The Change of Land Surface Temperature in Chongqing City 

**Figure 8:**
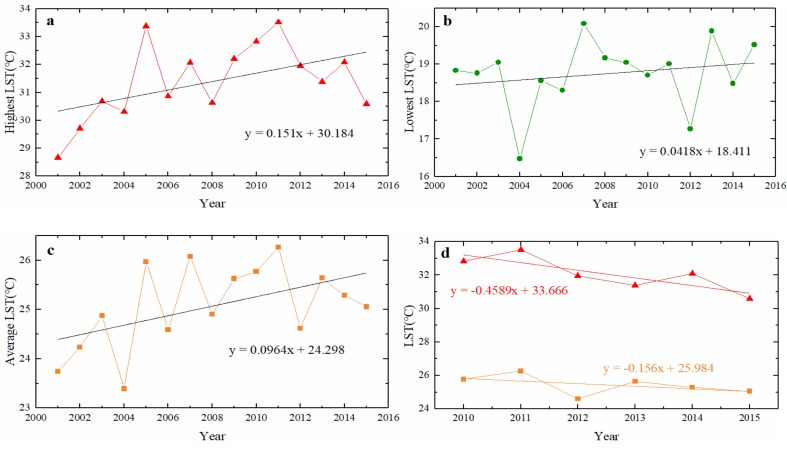
The change of LST (°C) in Chongqing City. (**a**), (**b**,**c**) represent the highest, lowest and average LST changes, respectively, from 2001 to 2015. (**d**) represents the changes of highest LST (the red line) and average LST (the orange line) from 2010 to 2015.

The water level of the TGRC was fully impounded at 175.0 m in 2010 [2]. This level may have some influence on Chongqing City (Figure 1). Therefore, this study addressed this region before and after 2010. The highest, lowest and average LST changes in Chongqing City showed an increasing trend from 2001 to 2015 with some fluctuations (Figure 8a–c). The LST value in 2000 was excluded due to the unavailable data before February resulted in significant increase of LST in the year. The LST in Chongqing City ranged from 18.83 °C to 28.66 °C in 2001, from 18.70 °C to 32.83 °C in 2010, and from 19.52 °C to 30.58 °C in 2015, respectively. The average LSTs were 23.74 °C in 2001, 25.76 °C in 2010 and 25.05 °C in 2015, respectively. The average and highest LSTs in Chongqing City showed increasing trends (0.2106 °C/a and 0.3481 °C/a) from 2001 to 2010, but decreasing trends (−0.156 °C/a and −0.4589 °C/a) from 2010 to 2015 (Figure 8d). 

### 3.5. Climate Change in the TGRC and Southwest China 

**Figure 9:**
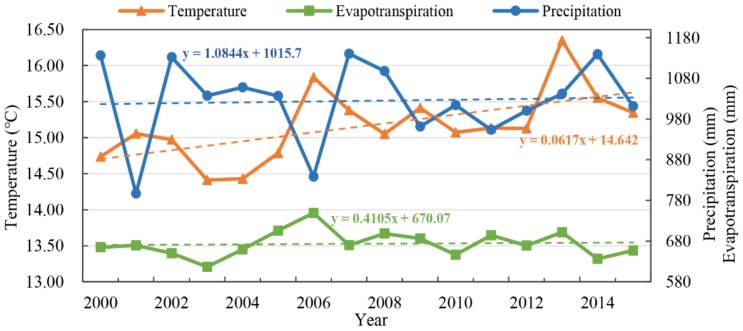
Changes in the air temperature, precipitation and evapotranspiration in the TGRC from 2000 to 2015.

The air temperature in the TGRC increased at a rate of 0.0617 °C/a over the past 16 years (Figure 9), the precipitation showed an increasing trend of 1.0844 mm/a and the evapotranspiration displayed an increase rate of 0.4105 mm/a. There were some large changes in 2006. The air temperature and evapotranspiration were at the peak value during the adjacent years, while the precipitation was at its bottom value. In addition, the air temperature had two more peak values in 2009 and 2013. What is more, the average value of evapotranspiration was relatively larger than that before 2003. In general, the air temperature showed dramatic change from 2000 to 2015, and the precipitation showed an increasing trend, while the evapotranspiration showed relatively smaller changes. 

**Figure 10:**
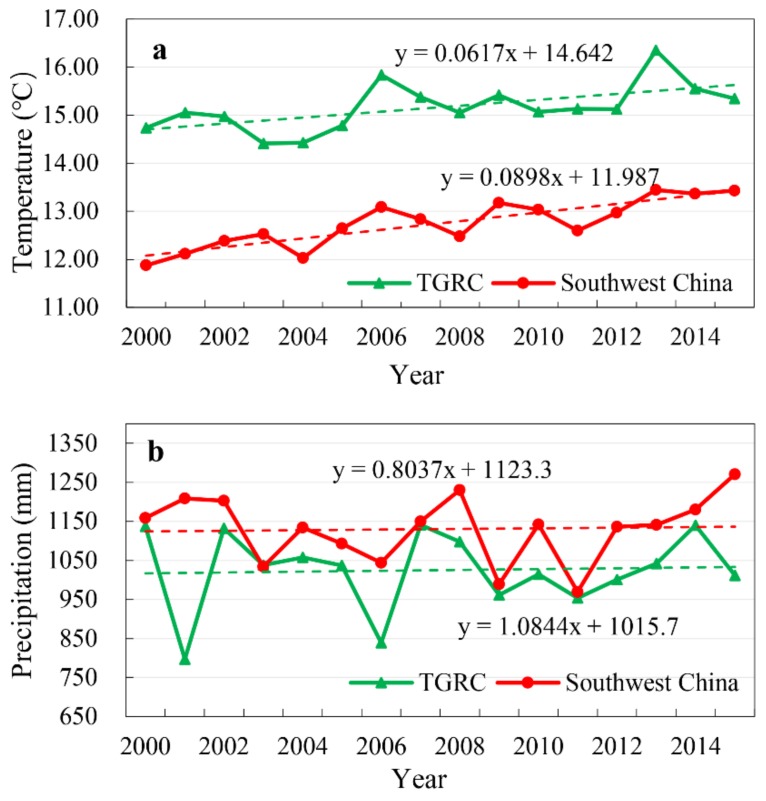

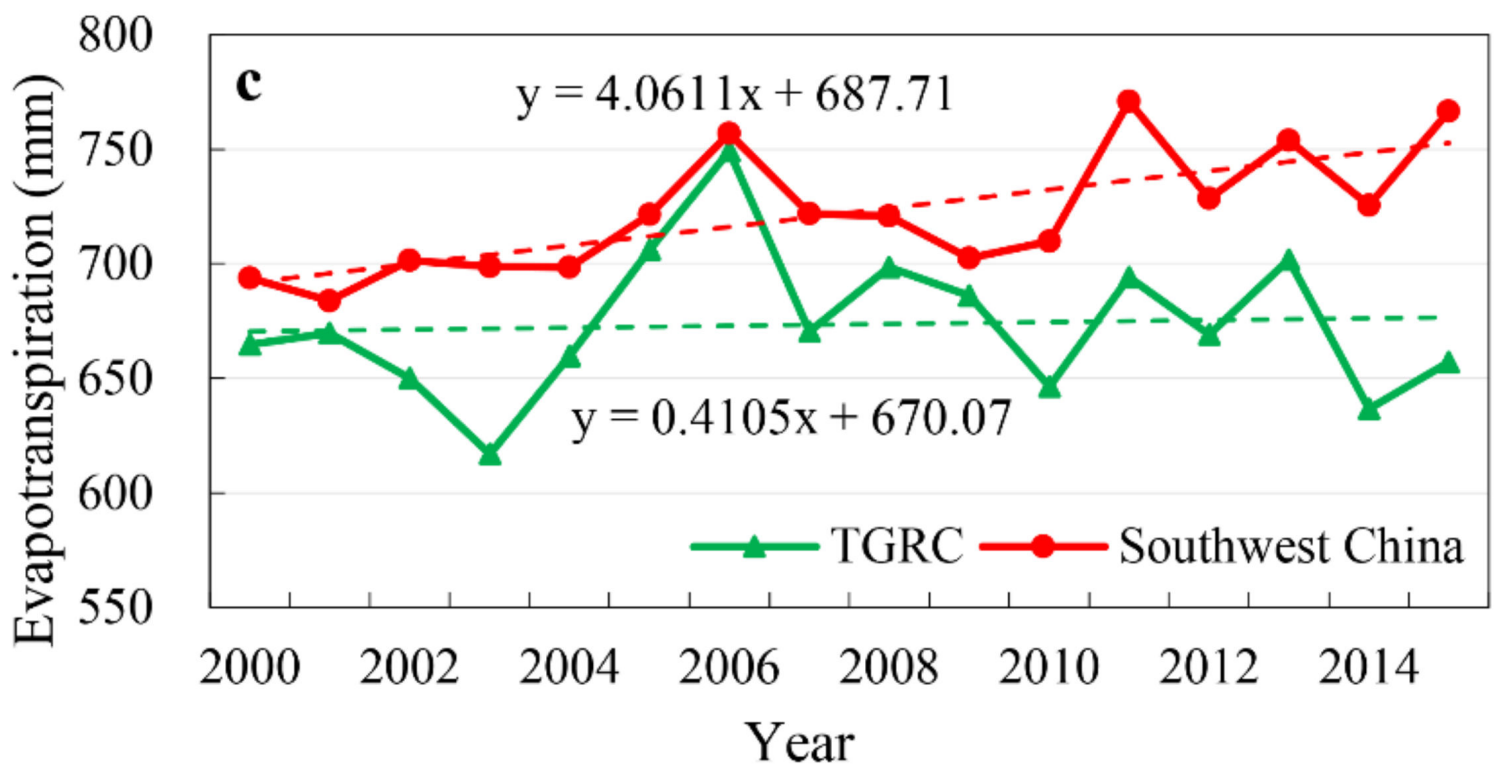
Variation of annual mean (**a**) air temperature, (**b**) precipitation, and (**c**) evapotranspiration in the TGRC and Southwest China from 2000 to 2015. The green line represents the variation in the TGRC, and the red line represents the variation in Southwest China.

In the period of 2000–2015, air temperature, precipitation and evapotranspiration in Southwest China all displayed an increasing trend (Figure 10). The air temperature in Southwest China showed a similar trend to that in the TGRC, which has some peaks in 2006, 2009 and 2013 (Figure 10a). There was significant relationship between air temperature in these two regions (*p* < 0.01, R^2^ = 0.569). The patterns of precipitation in the TGRC and Southwest China were generally similar (Figure 10b). Evapotranspiration showed similar increasing trends in these two regions, while more dramatic changes appeared in Southwest China (Figure 10c). 

### 3.6. Relations between Seasonally Integrated Normalized Difference Vegetation Index and Land Surface Temperature

**Figure 11:**
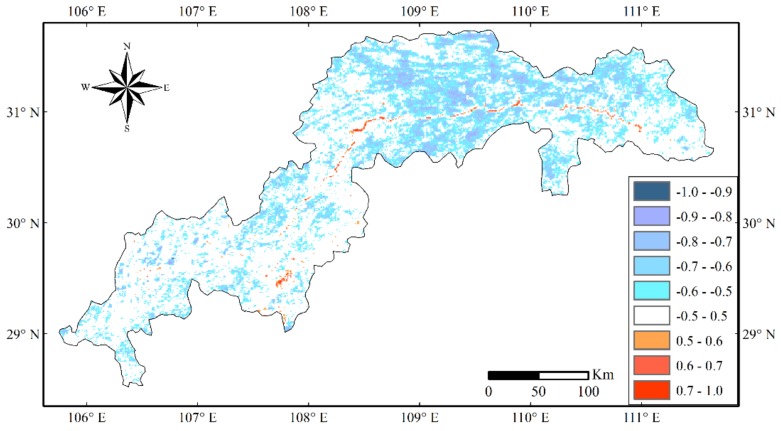
Spatial distribution of correlation coefficients between Seasonally Integrated Normalized Difference Vegetation Index (SINDVI) and land surface temperature (LST). The colored regions are characterized by a 95% confidence interval, while the areas are shown in white with *P* values higher than 0.05. Positive values mean positive correlation, while negative values mean negative correlation.

To quantify the spatial relation between the SINDVI and LST, the correlation coefficients of each grid cell were calculated. There was a negative correlation between the LST and SINDVI in most parts of the TGRC, especially in the tail and eastern part of the TGRC, primarily in the Chongqing City, Wuxi County, Yunyang County, Fengjie County, Wushan County and Xingshan County (Figure 11). However, there was also a positive relation between the LST and SINDVI primarily in the Yangtze River area where the LST was lower than the surrounding surfaces, no or less vegetation cover in the water area. 

### 3.7. Significant Change of Land Surface Temperature and Seasonally Integrated Normalized Difference Vegetation Index in Different Regions

**Figure 12:**
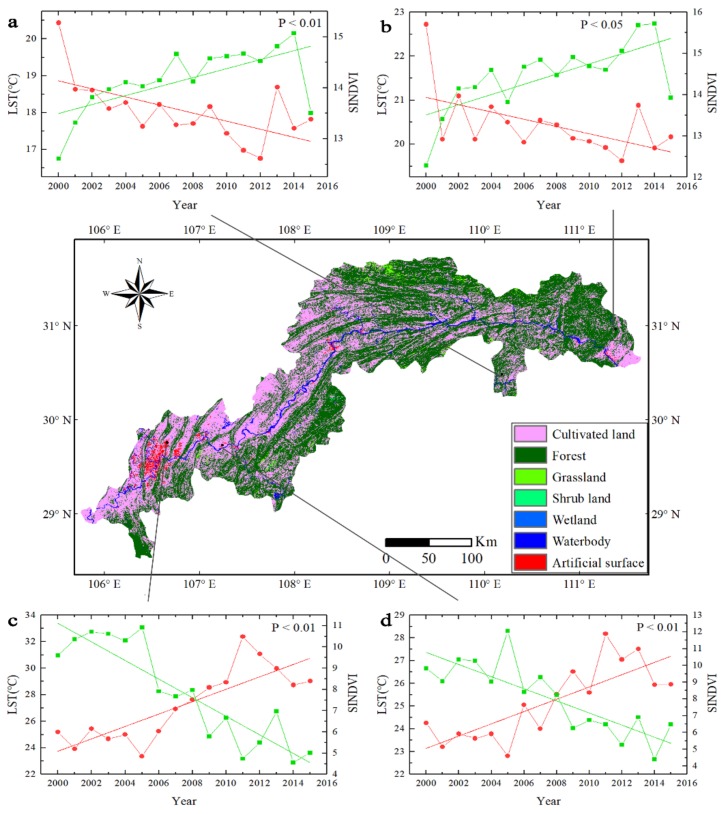
Time series change of the SINDVI (the green line) and LST (the red line) in four regions in the TGRC. The solid lines in green and red are the linear regressions of the time series data. The P values between the two specified variables are displayed in the upper right corner of each diagram box. The middle diagram is the land use map from 2015. The time series changes are the regional average values of grid cells area (1 km × 1 km). (**a**) Forest (30.48 °N, 110.18 °E). (**b**) Forest (30.96 °N, 111.38 °E). (**c**) Artificial surface (29.76 °N, 106.65 °E). (**d**) Artificial surface (29.73 °N, 107.24 °E).

The forest in the TGRC increased stably, while the artificial surfaces also increased rapidly over the research period. However, these area expansions occurred in different locations. For example, the forest was primarily distributed along the eastern TGRC, whereas the artificial surfaces appeared mainly in the tail of the TGRC. Hence, the typical distribution of land use types mentioned above was chosen and analyzed in four different regions (Figure 12). 

#### 3.7.1. Forest in the Eastern Three Gorges Reservoir Catchment

The eastern TGRC, specifically in Badong County (Figure 12a) and Yichang City (Figure 12b), was covered with a large amount of forest. Accordingly, the LST decreased. To investigate the change trends of LST and SINDVI, we calculated the time series regressions. The SINDVI increased with the slopes of +0.0897 (*R*^2^ = 0.4386) and +0.1236 (*R*^2^ = 0.4893) while LST decreased with the slopes of −0.1096 (*R*^2^ = 0.3778) and −0.0826 (*R*^2^ = 0.2934).

#### 3.7.2. Artificial Surface in the Western Three Gorges Reservoir Catchment

Chongqing City (Figure 12c) and Fuling District (Figure 12d) experienced a continuous increase in the urban area along with a decrease in vegetation coverage. SINDVI in these two regions shrank with the slope of −0.4378 (*R*^2^ = 0.8028) and -0.3614 (*R*^2^ = 0.6782) and LST grew with the slopes of +0.4673 (*R*^2^ = 0.6883) and +0.2701 (*R*^2^ = 0.6304). 

## 4. Discussion

### 4.1. Impacts of Land Use and Land Cover Change on Land Surface Temperature, Seasonally Integrated Normalized Difference Vegetation Index and Climate

In the last decade, one of the most significant effects of terrestrial ecosystem changes by human activities is the change in land use and land cover, which has greatly influenced the environment locally, regionally and globally [26,27]. The construction of the TGP, the expansion of urban areas and the ecological protection and restoration projects all have played an important impact on LULCC in the TGRC (Figure 5, Figure 6 and Figure 7). 

Our land use results were similar to some previous studies, while there are also some differences (Table 5). The source dataset, the land use type definition and classification may cause the difference between studies [20]. The land use results of Huang et al. [2] showed that there is no wetland but bare land in the TGRC. This is an obviously different land use classification. Moreover, they classified both forest and shrub land as forest; and the area sum of these two types of land use were similar (52.17% in this study versus 53.5% in their study). Meanwhile, the cultivated land, waterbody and artificial surface areas in this study were similar to their results (39.09% versus 40.4%, 2.80% versus 2.0%, 1.75% versus 2.80%, respectively). In addition, our results of waterbody (2.80%), cultivated land (39.09%) and grassland (3.16%) were similar to Guo et al. [52] waterbody (2.64%), cultivated land (37.38%) and grassland (2.41%). In spite of differences of land use type definition and the research time, these studies showed similar trends of increasing areas of forest, artificial surface and waterbody and decreasing areas of cultivated land and grassland. 

LULCC resulted in the changes of LST and vegetation coverage measured by SINDVI directly. LULCC was more significant in urban areas and forest area (Figure 12). In the forest, SINDVI showed an increasing trend due to the afforestation supported by the government in these areas, while the LST showed a decreasing trend due to processes such as vegetation transpiration and evapotranspiration [63]. In artificial surfaces, the SINDVI showed a decreasing trend, while the LST increased due to the rapid urbanization with the consequence of the vegetation reduction and resettlement area expansion [64]. Compared with artificial surfaces and forest, the correlation between SINDVI and LST in cultivated land is less clear. It could be due to the effect of agricultural activities on the crop SINDVI [65]. The periodical change in SINDVI in cultivated land caused the variation of LST and complicated the relationship between them. What is more, there was a negative relationship between LST and SINDVI in most areas of the TGRC, but a positive relationship in the Yangtze River where little vegetation appeared [66]. The LULCC has large impacts on vegetation cover and LST. The increase of forest area proved the effectiveness of environment protection policies (e.g., afforestation supported by government). However, the rapid urbanization should receive more attention due to the rapid loss of vegetation. It is clear that a better management of land use could minimize the impact of LULCC. 

The impacts of LULCC on climate change are complicated. There was a marked impact of LULCC on evapotranspiration. In 2003, the evapotranspiration experienced a dramatic change. The suddenly raised water level resulted in an expanded surface water area. Despite the influence of forest, precipitation, and air temperature [67], the main driver for evapotranspiration increase is the water surface expansion [68]. Previous studies showed an increasing trend of air temperature and precipitation in the TGRC, which was closely related to regional and global climate change [69,70]. Similarly, in this study, the change of climate factors in the TGRC was considered to be related to the climate change in the whole Southwest China area (Figure 10), especially air temperature and precipitation. It showed that LULCC has a certain level of impact on climate variation in the TGRC. However, it was consistent with regional climate change at a large scale. 

### 4.2. Causes for Changes in Land Use and Land Cover Change, Seasonally Integrated Normalized Difference Vegetation Index, Land Surface Temperature and Climate

The land use types in 2000, 2010 and 2015 showed large changes (Table 3 and Figure 5), including decreases in grassland and cultivated land, while increases in artificial surface, forest, shrub land, waterbody and wetland. The increase in artificial surfaces took place almost entirely in urbanized areas. This is mainly due to funding from the government and/or private enterprises to develop these areas for various industrial, commercial and residential purposes [71]. Hence, impermeable materials such as concrete were widely used in the construction of these buildings [72,73]. Additionally, the increase in forest primarily occurred in the eastern part of the TGRC, and this was ascribed to China’s national afforestation projects, for example Grain to Green (GTG) project. Cultivated land mainly lay in the northwest of the Yangtze River in the TGRC, especially the upper reaches; however, the decrease in cultivated land is a crucial problem for its importance of agriculture [74]. The reduction of cultivated land is mainly due to occupation by artificial surface, rising water level and poor management [75]. Grassland showed a dramatic decrease, and it was primarily submerged by the rising water level and converted to an artificial surface [42]. The expansion of waterbody was clearly accounted for by impoundment of the reservoir. Wetlands growing could be caused by the periodical change of inundation in the reservoir area [76]. The increase in shrub land could be mainly from the conversion of cultivated land [2]. 

LULCC directly influenced vegetation cover change. Vegetation cover measured by the SINDVI showed a greening trend, increasing by 3.209, in most parts of the study area, and mainly due to the afforestation projects and the better natural conditions (e.g., temperature and hydrothermal condition [77]) that were required for the vegetation growth [70]. Regions with higher SINDVI value were located in the tail and belly of the TGRC, such as Jiangjin County, Fengjie County and Yunyang County. The elevations of these counties are relatively high and covered with forest. Therefore, they are less affected by human activities and ecological restoration programs resulted in an increase of SINDVI [2,78]. However, regions with decreasing SINDVI mainly appeared in the urbanized area, particularly in Chongqing City, Yichang City, Fuling District, Wanzhou District and their neighboring regions (Figure 6). Similarly, a study also found a continuous increase of buildings, roads and industrial areas, but a decrease of forest and grassland in these regions [52]. This is the main reason for the decrease of SINDVI in these areas. 

The LST decreased by 0.253 °C in the TGRC in the period of 2001–2015. The LST showed a decreasing trend mainly in non-urban areas located in the south bank of the Yangtze River and the eastern part of the TGRC, which might be caused by continuous afforestation [79]. Many studies found that LULCC can influence LST [80]. In particular, expansion of urban areas can increase LST by creating a so called urban heat island [81,82]. In the TGRC, the LST showed a large increase in urbanized areas, such as Chongqing City, Yichang City, Fuling District and Wanzhou District. This might be due to the increased residential, commercial, and industrial areas, as well as declined vegetation [83,84]. The highest LST, lowest LST and average LST all showed an increasing trend in Chongqing City from 2001 to 2015. However, the highest LST and average LST showed a smaller decreasing trend when the water level rising to 175.0 m after 2010, probably due to increasing amounts of water around the city and a large buffer of waterbody to constrain the rise in temperature [30,85]. 

LULCC has certain impacts on climate variation in the TGRC. Some studies found the effect of increased water area on climate change. In particular, the effects were locally within a buffer along the reservoir. For example, the evapotranspiration increased markedly after the operation of the Three Gorges Hydropower Project in 2003 with the consequence of huge expansion of water area [68]. Meanwhile, climate in the TGRC was also influenced by vegetation cover in the catchment and large scale climate change in Southwest China [70,86]. It is more important, at a larger scale, that the atmospheric circulation in Southwest China influenced the precipitation in the TGRC [70]. In general, the air temperature variation in the TGRC was consistent with the change trend in Southwest China, which is related to the change trend of global air temperature [87]. For example, the droughts in 2006, 2009, and 2013 in the TGRC were considered to be more related to the climate change in Southwest China, and less related to the local factors in the TGRC [88,89,90]. Consequently, the climate variation in the TGRC was influenced more dramatically by climate change at a large scale, rather than local factors in the catchment.

### 4.3. Mitigate the Impact of Environmental Change in Three Gorges Reservoir Catchment

Managing land use and mitigating the potential impacts are essential for the sustainable development in this area [91]. During our research period, the artificial surfaces increased rapidly, while the grassland, cultivated land and forest have decreased continuously. The disorder urban expansion has led to the waste of land resources, traffic congestion and other social problems [92,93]. Therefore, more science-based land resource management regulations are clearly needed. China’s government has proposed several environmental protection programs [78]. It is urgent for local governments to take appropriate measures to avoid unnecessary construction of houses [94]. Worse, the cultivated land decreased by 1,182.75 km^2^ from 2000 to 2015. As a national problem, loss of cultivated land should be reversed with strict protection [95]. More importantly, the strict enforcement of the laws and regulations is the key for the sustainable using of land resource [96]. 

### 4.4. Advantages of Data Processing and Visualizing using Google Earth Engine

The cloud-based computation ability, and archived Landsat, MODIS and other social-economic and geographical data in the GEE present many advantages for the spatiotemporal studies [41,42]. In addition, the integrated APIs and data archive in GEE have facilitated researches. What is more, instead of downloading massive amounts of data to a local server or personal computer, we could simply use, process, analyze and export results on GEE. Based on GEE, we found that vegetation restoration and afforestation led by the government was very effective for improving vegetation coverage [97]. In addition, utilizing the GEE platform, at a fast speed, we have monitored and tracked the vegetation cover, LST change, climate change influenced by LULCC in the TGRC. More importantly, our research method can be easily applied in long-term monitoring on vegetation cover change, LST change, and LULCC in the TGRC, which could provide more timely information of the environment changes in the region. 

### 4.5. Limitations and Future Research

Similarly to many other studies, there are limitations in the current study. The land use maps at a 30 m spatial resolution from 2000 and 2010 were acquired from the NGCC; however, they only provide the products for these two baseline years. To investigate the land use types after 2010, we used Landsat 8 images with a high classification accuracy of 95.02%. The land use map in 2000 and 2010 were obtained by clipping the GlobeLand30 raster data using the TGRC vector file (shape format), and then this vector file was imported to GEE as the study area boundary and executed the classification. However, a certain number of triangle images were not classified due to the jagged effect between vector data and raster data, and the total area of these triangles were about 22.17 km^2^ (0.0384% of the TGRC area). Therefore, an adjustment of 3.167 km^2^ (22.17/7) was added to each land use types in the TGRC to meet the total area of 57,669.55 km^2^. 

The MODIS NDVI product and the LST product both lack data before February 2000 and this may erode time-series regression of these variables. In terms of climate variation in the TGRC, some authors argued the marked influence of large dam on the local climate [98,99]. In the future, more factors and models embedded in the GEE platform including albedo, soil moisture, the population, climatic model and others, will increase our understanding of LULCC and its impacts on LST, vegetation cover and climate change in the area of the world’s largest hydropower plant. 

## 5. Conclusions

In this study, the LULCC in the TGRC were investigated from 2000 to 2015, and its impacts on vegetation coverage, LST and climate were analyzed. Our results showed the forest, waterbody, artificial surface, shrub land, and wetland expanded, while the grassland and cultivated land shrank. The large LULLC in the TGRC has resulted in the change of vegetation cover and LST. A total of 93.23% of SINDVI in the TGRC showed an increasing trend from 2000 to 2015, indicating a clear expansion of vegetation coverage in the TGRC. A total of 88.76% of LST in the study area displayed a decreasing trend. Specifically, Chongqing City has the highest LST and the lowest vegetation coverage in the whole area. The LST and SINDVI values varied between the different land use types. In general, the multiyear SINDVI and LST have significantly negative correlations at most areas of the TGRC. The air temperature, precipitation, and evapotranspiration showed increasing trends from 2000 to 2015 in the TGRC. Similarly to other areas in Southwest China, the LULCC has certain impacts on climate change. In addition, thanks to the environmental protection measures, the forest area increased stably in the TGRC, and it increased more obviously in the eastern part of the TGRC, such as Wushan County, Wuxi County, and Fengjie County, which proved that a better management of land use changes is crucial for environment change in the TGRC. However, the grassland and cultivated land decreased continuously in Chongqing City and its neighboring regions, Wulong County, Zhong County, and Fengdu County. Clearly, more effective measures are still needed for proper management of land use in the catchment, particularly in Chongqing City, Wulong County, and Zhong County. Our study showed the advantages of using GEE to analyze the spatiotemporal dynamics of the LULCC, vegetation cover, LST, and climate for a long time-series, and highlighted the importance of environmental protection for the sustainable development in the catchment. 

## Figures and Tables

**Table 1:** Summary of sampling sites in the Three Gorges Reservoir Catchment (TGRC).

	Cultivated Land	Forest	Grassland	Shrub Land	Wetland	Water Body	Artificial Surface
TSs	854	393	146	39	29	674	349
VSs	366	168	62	17	12	289	150
TN	1220	561	208	56	41	963	499

TSs represent training samples, VSs represent validation samples, and TN represents the total number.

**Table 2:** Detailed information on the Landsat 8 Operational Land Imager (OLI) images.

Band Number	Spectral Range (µm)	Spatial Resolution (m)	Band Name
1	0.435–0.451	30	Coastal/Aerosol
2	0.452–0.512	30	Blue
3	0.533–0.590	30	Green
4	0.636–0.673	30	Red
5	0.851–0.879	30	NIR
6	1.566–1.651	30	SWIR1
7	2.107–2.294	30	SWIR2
8	0.503–0.676	15	Pan
9	1.363–1.384	30	Cirrus
10	10.60–11.19	100	TIR-1
11	11.50–12.51	100	TIR-2

**Table 3:** Land use change in the TGRC from 2000 to 2015.

Class Name	Area km^2^ 2000	Area % 2000	Area km^2^ 2010	Area % 2010	Area km^2^ 2015	Area % 2015
Artificial Surface	509.90	0.88	730.57	1.27	1008.96	1.75
Forest	26,998.80	46.82	28,269.85	49.02	29,102.28	50.46
Waterbody	776.10	1.35	954.73	1.66	1616.45	2.80
Wetland	50.97	0.09	29.33	0.05	591.65	1.03
Shrub land	312.59	0.54	318.21	0.55	985.45	1.71
Cultivated land	23,727.43	41.14	23,687.01	41.07	22,544.68	39.09
Grassland	5293.76	9.18	3679.85	6.38	1820.08	3.16
Total	57,669.55	100	57,669.55	100	57,669.55	100

**Table 4:** Proportion of areas with different vegetation change in the TGRC from 2000 to 2015.

	Decreasing	No Significant Change	Increasing
Value range	<−0.1	−0.1–0.1	>0.1
Area percentage	4.53%	1.24%	93.23%

**Table 5:** The comparison of land use type’s areas percentage in the TGRC from various studies.

Class Name	This Study	Huang et al.	Guo et al.
Temporal (Year)	2015	2015	2013
Artificial Surface	1.75	2.80	2.96
Forest	50.46	43.0	54.55
Waterbody	2.80	2.0	2.64
Wetland	1.03		
Shrub land	1.71	10.5	
Cultivated land	39.09	40.4	37.38
Grassland	3.16	0.3	2.41
Bare land		1.1	
Unused land			0.03

Note: The blank represents no category in the classification system in the corresponding reference.
